# The Relationship between the Morphology and Elasticity of Natural Rubber Foam Based on the Concentration of the Chemical Blowing Agent

**DOI:** 10.3390/polym13071091

**Published:** 2021-03-30

**Authors:** Supitta Suethao, Saree Phongphanphanee, Jirasak Wong-ekkabut, Wirasak Smitthipong

**Affiliations:** 1 Specialized Center of Rubber and Polymer Materials in Agriculture and Industry (RPM), Department of Materials Science, Faculty of Science, Kasetsart University, Bangkok 10900, Thailand; supitta.sue@gmail.com (S.S.); fscisrph@ku.ac.th (S.P.); fscijsw@ku.ac.th (J.W.-e.); 2 Department of Physics, Faculty of Science, Kasetsart University, Bangkok 10900, Thailand; 3 Office of Research Integration on Target–Based Natural Rubber, National Research Council of Thailand (NRCT), Bangkok 10900, Thailand

**Keywords:** rubber foam, morphology, elasticity, thermodynamics, chemical blowing agent

## Abstract

Concentrated natural latex was used to produce a rubber foam that is porous, elastic and well ventilated. The mechanical properties can be either soft or firm, depending on the formulation of the latex used. Briefly, concentrated natural latex was mixed with chemical agents to make the rubber foam on a laboratory scale using the Dunlop process. In this work, we changed the concentration of the chemical blowing agent in the latex. The morphological properties of the rubber foam were characterised using scanning electron microscopy, and the mechanical properties, or elasticity, were studied using compression experiments and the Mooney–Rivlin calculation. The results show that the concentration of the chemical blowing agent affects the morphological properties of the rubber foam but not the mechanical properties, indicating the heterogeneous structure of the rubber foam. The thermodynamic parameters (∆*G* and ∆*S*) and the internal energy force per compression force (*F*_u_/*F*) of the rubber foam with various amounts of chemical blowing agent were also investigated. This study could be applied in the foam industry, particularly for pillow, mattress and insulation materials, as the present work shows the possible novel control of the morphological structure of the rubber foam without changing its mechanical properties. The difference in cell sizes could affect the airflow in rubber foam.

## 1. Introduction

Natural rubber foam (NR foam) is a good example of a long-chain polymer with great elasticity. It also possesses excellent mechanical properties with different morphological properties. NR foam is a lightweight material with many porous structures, indicating a good compromise between strength and weight [1,2]. NR foam is prepared from both concentrated natural latex and non-latex rubber [3,4]. It has been used for many years to produce many products, such as mattresses, pillows, dolls, cushions, flooring and upholstery foam [5,6,7,8]. Many processes are used in rubber foam production, but the most popular process is the Dunlop process. The Dunlop process is a trendy process used to produce foam rubber with open-cell morphology [9].

The previous works focused on varying concentrations of sodium bicarbonate as a chemical blowing agent in natural rubber foam [10,11]. However, these works studied different types of natural rubber: concentrated natural latex and dried natural rubber. They found that the relative density of both types of NR foam decreased with increased amounts of chemical blowing agent. The cell density increased with increased amounts of the chemical blowing agent. Moreover, the compression strength of NR foam from latex tended to decrease with increasing chemical blowing agent concentrations, while the NR foam from dried rubber with higher chemical blowing agent concentrations absorbed more energy. Since more of the air bubble from chemical blowing agent is presented, the rubber foam reveals softer. The existence of the air bubble in rubber foam exhibits the energy-absorbing characteristic of foam.

The effects of cell size and the interconnected matrix (or cell wall thickness) on the Young’s modulus of closed-cell foams made of styrene–acrylonitrile were studied using Laguerre models. The results showed that the stiffness decreased with increasing cell size, so the stiffness of polymer foams related to the cell size distribution and interconnected matrix was investigated [12,13]. The influence of the foaming condition on the cellular structure of poly(methyl methacrylate) (PMMA) foam has been studied by varying the CO_2_ pressure and temperature. The production of PMMA foams with control structures was prepared by using CO_2_ as the physical blowing agent. The results showed that the largest void fraction of PMMA foam, with a cell size of less than 5 μm, could be produced, improving the thermal and mechanical properties of PMMA foam.

The main properties of rubber foam are low weight, high buoyancy, cushioning capability, flexibility, thermal insulation, acoustic insulation and impact dampening [11,14]. Because of this great variety of properties, rubber foam can be used in many applications. Many researchers have studied the factors affecting these properties. However, little work has been done to understand the effect of the chemical blowing agent on the morphology and elasticity of foams. A thermodynamic investigation of rubber foam from concentrated natural latex is particularly lacking. The novelty of this work is to reveal the relationship of the morphology, elasticity and thermodynamic properties of rubber foam to the effects of the chemical blowing agent, developing a semi–empirical model to design the NR foams with different microscale morphological structures but unchanged macroscale mechanical properties.

## 2. Materials and Methods

### 2.1. Materials

Commercial high ammonia (HA) concentrated natural latex was obtained from Num Rubber and Latex Co., Ltd., Trang, Thailand. An aqueous solution of 10% potassium oleate (Po) and aqueous dispersions of 50% sulphur, 50% zinc–N–diethyldithiocarbamate (ZDEC), 50% zinc–2–mercaptobenzothiazole (ZMBT), 50% antioxidant (Wingstay L), 50% zinc oxide (ZnO), 33% diphenylguanidine (DPG) and 12.5% sodium silicofluoride (SSF) were supplied from Thanodom Technology Co., Ltd., Bangkok, Thailand.

### 2.2. Preparation of Compounded Latex

To prepare the compounded latex, the ammonia in the concentrated natural latex was evaporated by blending at a low speed (80 rpm) for 1 min. Then, various concentrations of Po in an aqueous solution were added, and the blender speed was increased to 160 rpm for 8 min or the time needed to achieve a bubble volume of 3.5 L. Next, the aqueous dispersions of sulphur, ZDEC, ZMBT and antioxidant (Wingstay L) were added, and the speed was decreased to 80 rpm for 1 min. After that, the aqueous dispersions of ZnO and DPG were added and mixed for a further 1 min. Finally, the aqueous dispersion of SSF was added and mixed until the gel-forming state was reached (Table 1 and Table S1).

### 2.3. Preparation of Rubber Foam

To prepare the rubber foam, the compounded latex was poured into a stainless-steel mould, which was 50 mm in width, 50 mm in length, and 25 mm in height. Then, the mould was kept at room temperature for 45 min to stabilise the bubbles. Next, the mould was moved to a 90 °C oven for 2 h to vulcanise the rubber foam. Finally, the rubber foam was removed from the mould, washed, and dried in an oven at 70 °C for 4 h.

### 2.4. Characterisation

The morphological properties of the foam samples were evaluated by scanning electron microscopy (SEM, Quanta 450 FEI, Eindhoven, Netherlands). The rubber foam (surface and bulk) was cut into small samples and coated with gold in a special coater (Polaron Range SC7620, Quorum Technologies Ltd., Kent, UK). ImageJ software [15] was used to analyse SEM images and calculate cell sizes and porosity. Cell density can be calculated following the previous work [16]. Foam density was measured in kg/m^3^ using the weight at a constant volume of foam sample.

Chemical functional groups of the foam samples were analysed by attenuated total reflection–Fourier transform infrared spectroscopy (ATR–FTIR, VERTEX 70, Bruker, Billerica, MA, USA) with a Ge crystal probe at 500–4000 cm^−1^.

Mechanical properties of foam samples in compression mode were characterised by a texture analyser (TA.XT Plus, Stable Micro Systems, Godalming, Surrey, UK) using a 100 mm diameter platen probe with a speed of 0.1 mm/s and compressed at 75% from the foam surface at room temperature. Moreover, we applied the Mooney–Rivlin equation [17] at the stress (σ) and compression limit (*λ*) of foam samples with *C*_1_ and *C*_2_ as constant values as follows:

(1) $$\frac{\sigma }{\left(\lambda -\frac{1}{\lambda^{2}}\right)}=2C_{1}+2C_{2}\frac{1}{\lambda }$$

The crosslinking density and volume fraction of the rubber foam samples were determined by the swelling method and the Flory–Rehner equation [18,19,20]. We also used the Flory–Huggins equation to calculate the change in Gibbs free energy (∆*G*) and entropy (∆*S*) as follows [21,22]:

(2) $$\Delta G=RT\left[\ln\left(1-V_{r}\right)+V_{r}+V_{r}^{2}X\right]$$

(3) $$S=\frac{\Delta G}{T}$$

where *V*_r_ is the volume fraction of the foam sample in the rubber network, *χ* is the parameter between the foam sample and the solvent interaction (defined as 0.43 + 0.05 *V*_r_) [23], *R* is the ideal gas constant (8.3145 J/mol·K), and *T* is the test temperature (298.15 K).

The foam sample was evaluated using a texture analyser (TA.XT Plus, Stable Micro Systems, Godalming, Surrey, UK) using a speed of 0.1 mm/s. The sample was held for 10 min to ensure a constant temperature. The foam sample was then compressed from 10% strain to 70% strain at different temperatures (298.15, 308.15, 318.15, 328.15 and 338.15 K). Then, the force–temperature relationship graph during the deformation process was determined, and the parameters obtained were used to calculate the internal energy force (*F*_u_) from the intercept at 0 K and the entropy force (*F*_s_) from the slope multiplied by the temperature.

## 3. Results and Discussion

The effect of the chemical blowing agent (potassium oleate) on foam rubber was studied in this work by varying the potassium oleate concentration. In this study, foam rubber was prepared with varying concentrations of the chemical blowing agent (0.90, 1.15, 1.40 and 1.65 phr or parts per hundred of rubber as in Table S1) corresponding to the control - 45% Po, the control - 30% Po, the control - 15% Po and the control. Thus, the chemical blowing agent concentration at 1.65 phr is the control formula (Table S1), and the concentration has been decreased to study the effect of the chemical blowing agent on the morphological properties and elasticity of the rubber foam.

### 3.1. Morphological and Physical Properties

**Figure 1:**
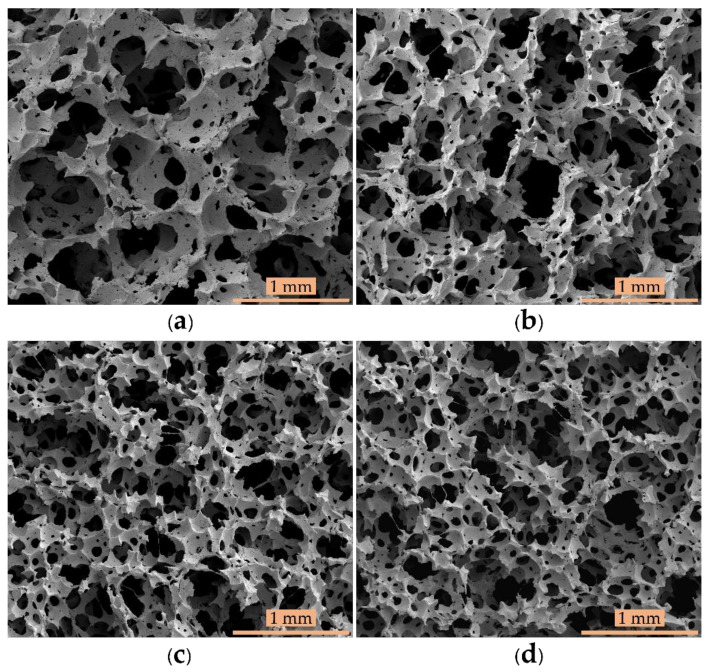
SEM images of foams created with various concentrations of chemical blowing agent at 150× magnification: (**a**) control foam, (**b**) control - 15% Po foam, (**c**) control - 30% Po foam, and (**d**) control - 45% Po foam.

The morphological properties of all the foam samples are illustrated in Figure 1, showing the effect of the chemical blowing agent (potassium oleate) on morphology. There were two structures—pores or cells—representing the void space and cell wall thickness, making up the interconnected foam matrix. The cells were generally interconnected with each other, an open–cell structure. This structure comes from the Dunlop preparation method, which mixes the latex at high speed, generating different sizes of gas bubbles [24,25]. The open–cell structure with connected foam cells can occur with gas bubbles of either the same or different sizes. When two gas bubbles are near each other, the gas can transfer from the smaller bubble to the larger bubble, resulting in the bubbles merging to become an open-cell structure [26,27]. As the chemical blowing agent concentration decreased, micrographs showed more interconnected foam structures with smaller cell sizes than those of the control sample. However, the morphological structure of polymer foam can be made either open cell or closed cell [28]. William and Wrobleski investigated the effect of surfactant–stabilised HIPE (high internal phase emulsions) on polyHIPE foam properties [29]. They found that this polymer foam has a closed–cell structure at a surfactant content below 7 vol%, but beyond this concentration, the polymer foam has an open–cell structure.

**Figure 2:**
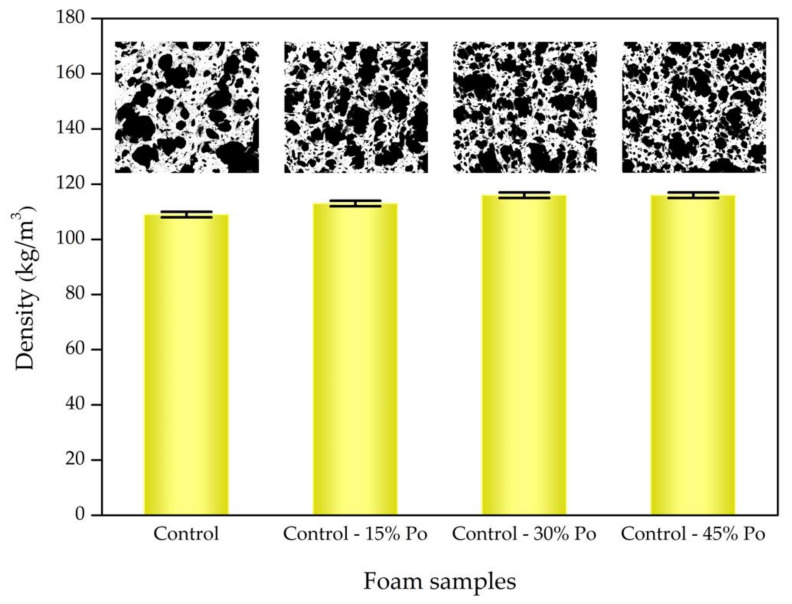
SEM images analysed by ImageJ software and foam density as a function of foam samples with different concentrations of the chemical blowing agent.

Figure 2 shows SEM images of all the foam samples processed using the ImageJ software, where the black colour indicates the foam cell and the white colour means interconnected matrix. We calculated the average cell size and porosity of the foam samples using these images, and the results are shown in Table 2. We found that the average cell size of the foam samples decreases with decreasing potassium oleate concentration because smaller gas bubbles were generated. The overall porosity of the foam samples seems to increase with decreasing chemical blowing agent, indicating the effect of smaller and larger gas bubble distributions in the foam matrix. Low concentrations of chemical blowing agent in foam samples generated small gas bubbles resulting in high cell density. The morphology of surface and bulk foam samples was similar, based on the heterogeneous cell size generated by the Dunlop process. The relationship between the average cell size and the blowing agent concentration in this work is in good agreement with previous research [30]. Both works used chemical blowing agent concentrations below 3 wt%. However, the opposite relationship of cell size and blowing agent concentration was reported in polysulfone (PSU) foam [31]: the average cell size decreased with an increase in the physical blowing agent, a CO_2_ feed at high concentrations in the PSU. The morphology of the polymer foam depends on the foam preparation method and the type of blowing agent. The computer models based on self-consistent field theory (SCFT) and classical nucleation theory (CNT) propose a model of the best polymer foam with simultaneous small cell size and high cell density [32].

To investigate the physical properties of rubber foams, foam density results are presented in Figure 2. Lower blowing agent concentrations free the gas to escape through the foam surface, allowing the foam to condense more, and consequently, foam with a slightly higher density is produced. However, it seems that a constant foam density is reached at blowing agent concentrations below 70% or 1.15 phr. The rubber foam with chemical blowing agent concentrations above 1.65 phr has a foam density similar to the control sample (chemical blowing agent concentration of 1.65 phr). This result tends to agree with the foam densities of various chemical blowing agent (i.e., sodium bicarbonate) concentrations in dried natural rubber [11].

The chemical structures observed in the ATR–FTIR spectra of all the foam samples are not significantly different (Figure S1). They contain peaks at 3015–2970 cm^−1^ from C–H vibrations (CH_3_ and CH_2_) and 1672 cm^−1^ from C = C double bonds in the natural rubber molecule. An absorption peak between 935 and 1171 cm^−1^ belongs to C–S stretching, which confirms crosslinking between sulphur atoms and natural rubber molecules and that the peak at 840 cm^−1^ is associated with C = C wagging in natural rubber [24,34,35].

### 3.2. Physical and Chemical Elasticity

The elasticity of rubber foam can be derived assuming that mechanical compression relates to the physical elasticity and that swollen rubber foam in a good solvent relates to the chemical elasticity. Both types of elasticity can be applied to evaluate the thermodynamic parameters in different ways.

**Figure 3:**
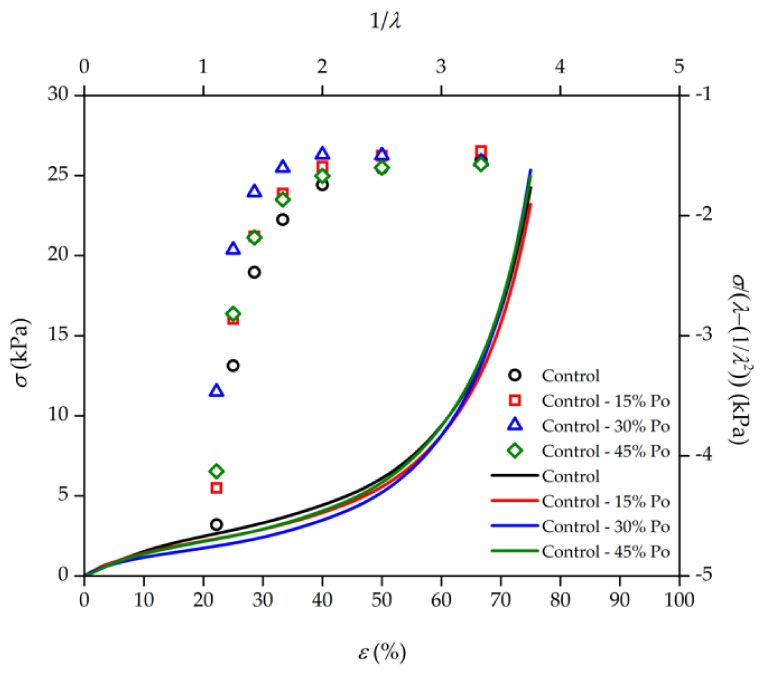
Results of the mechanical properties of rubber foam in two forms: (i) stress (σ)–strain (ε) curves of foam samples with various concentrations of chemical blowing agent in lines; (ii) the Mooney–Rivlin equation [17] for rubber foam in the unfilled symbols. The relative scatter on the results is estimated to be about 5%.

Figure 3 shows the mechanical properties of rubber foam in two ways. First, the compression stress (*σ*)–strain (*ε*) curve of rubber foam samples is presented. Rubber foam can be deformed until around 50% strain before non–linear elastic collapse caused by the elastic buckling of cell walls occurs. The elastic collapse stress and the post-collapse behaviour depend on the open–cell structure, which shows a long flat plateau in the stress–strain curve [36]. Second, at large compression strains, the opposing walls of the cells crush together, and the cell wall material itself is compressed. This is the strain at which all the pore space has been collapsed, otherwise known as the densification regime [37]. For uncrosslinked polymer foam, the phenomenon of strain–induced crystallisation can be accounted for by these mechanical properties at high strains [38].

We found no significant differences in the macroscopic compression stress–strain curves in any foam samples (Figure 3). As discussed before, higher chemical blowing agent concentrations lowered the foam density since more gas was generated. However, the mechanical properties from the stress–strain curve were similar, indicating the heterogeneous structure of the rubber foam produced with the Dunlop method [39,40]. The control sample had a large cell size, and the matrix had a small cell size distribution. When the chemical blowing agent concentration was reduced, the cell size became smaller, and the cell density increased. However, the porosity percentage was only slightly different. This similarity explains why the mechanical properties of all the samples were similar. Therefore, the morphological properties of rubber foam with a heterogeneous structure are more sensitive to the concentration of the chemical blowing agent than the mechanical properties in compression mode. The mechanical properties of rubber foam depend not only on the morphological properties [41] but also on the interconnected foam matrix [33]. For the same polymer concentration without filler, the mechanical property (or storage modulus) of poly(methyl methacrylate) (PMMA) foam can differ with the difference of cell sizes between nanosized and microsized foams [41].

Figure 3 also presents the compression curves based on the extension of the Mooney–Rivlin equation [17], which is why our compression result has the opposite trend to the Mooney–Rivlin results. From the Mooney–Rivlin equation, we found similar crosslinking densities for all foam samples at high compression strains (or high 1/***λ***), while, at low compression strains (or low 1/***λ***), the control sample with the highest amounts of chemical blowing agent had the lowest stress compared to that of the foam samples with lower amounts of the chemical blowing agent. This is remarkably interesting considering the difference in compression between low and high strains, which is related to the difference between morphological structures. At low strain, the NR foam cells are buckled, whereas the foam cells are completely collapsed at high strains. Therefore, we can find the different mechanical properties of NR foams based on the Mooney–Rivlin equation [17] at low strain, but this mechanical property of NR foams is quite similar at high strains.

Table 3 presents the *C*_1_ and *C*_2_ of our crosslinked NR foam samples in compression mode based on the Mooney–Rivlin equation [17]. Generally, 2*C*_1_ relates to the shear modulus (*G*), as in Equations (4) and (5), and 2*C*_2_ relates to the shear modulus of polymer entanglement (*G*_e_). We found that our *C*_1_ is in reasonable agreement with the literature reviews [42,43], confirming that crosslinked solid rubber possesses a high shear modulus and high *C*_1_ compared to uncrosslinked solid rubber. The ratio *C*_2_/*C*_1_ from our result is in good agreement with the literature [42,43]. The shear modulus (*G*) can be written as follows:

(4) $$G=f_{c}G_{c}+\emptyset \left(\lambda \right)G_{e}$$

(5) $$\emptyset \left(\lambda \right)=\frac{1}{\lambda }$$

where *f*_c_ is the function of network defects, *G*_c_ is the shear modulus of the polymer network, and *G*_e_ is the shear modulus of polymer entanglement.

The chemical elasticity can be studied using the crosslinking density and volume fraction of the rubber of various foam samples, which has only small differences (Table 4) because of the effect of the chemical blowing agent on the morphological properties of the foams. These results relate to the change in the Gibbs free energy (∆*G*) in negative values and the change of entropy (∆*S*) in positive values, which correspond to the spontaneous thermodynamic favourability.

**Figure 4:**
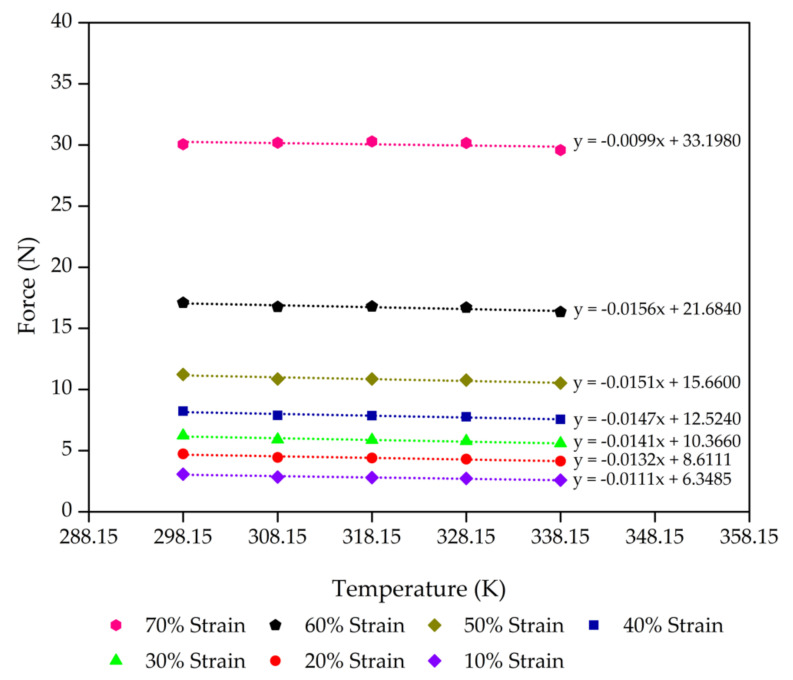
Compression force as a function of the temperature of the control sample from 10% to 70% compression strain with a minimum linear regression (R^2^) = 0.9 at each strain. The scatter on the results is on the order of the size of the data points.

**Figure 5:**
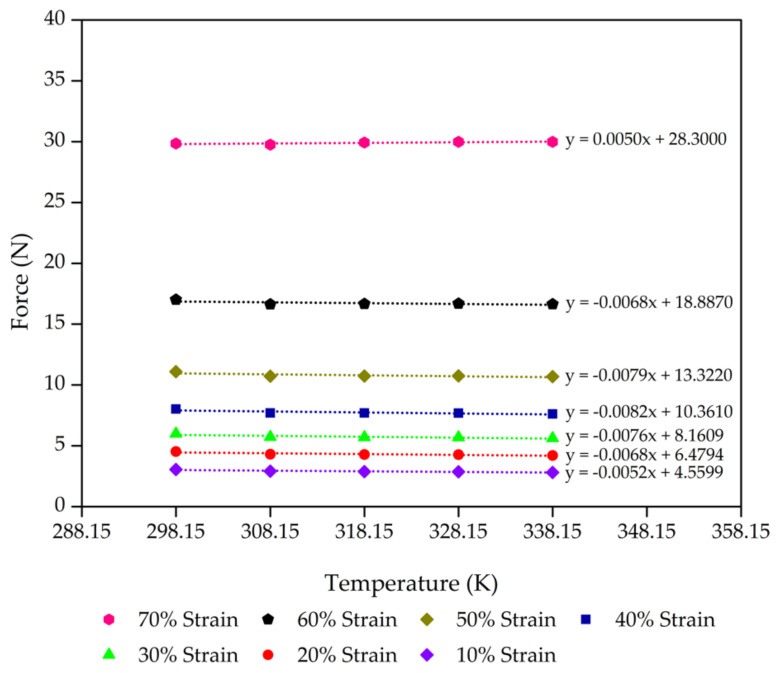
Compression force as a function of the temperature of the control - 15% Po sample from 10% to 70% compression strain with a minimum linear regression (R^2^) = 0.9 at each strain. The scatter on the results is on the order of the size of the data points.

**Figure 6:**
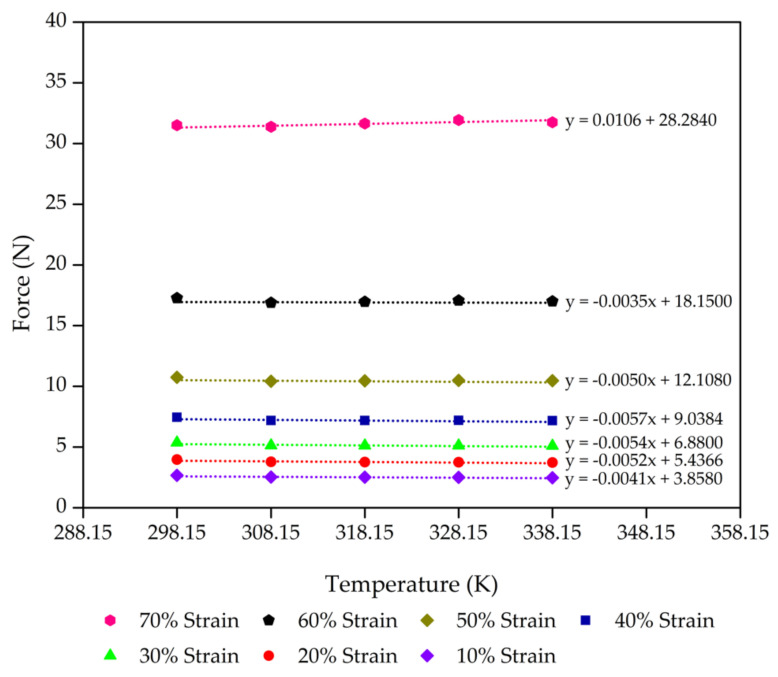
Compression force as a function of the temperature of the control - 30% Po sample from 10% to 70% compression strain with a minimum linear regression (R^2^) = 0.9 at each strain. The scatter on the results is on the order of the size of the data points.

**Figure 7:**
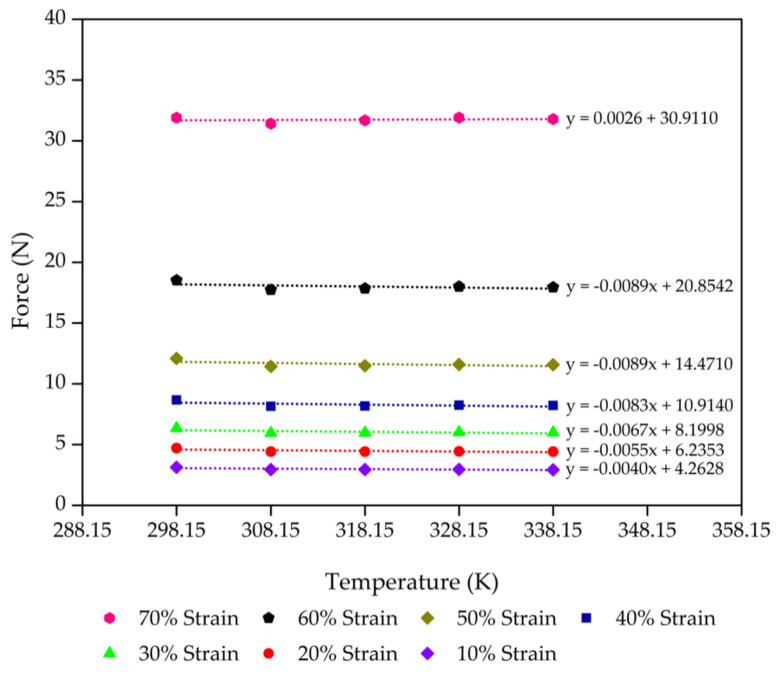
Compression force as a function of the temperature of the control - 45% Po sample from 10% to 70% compression strain with a minimum linear regression (R^2^) = 0.9 at each strain. The scatter on the results is on the order of the size of the data points.

Consideration of the thermodynamics of the process of extension elasticity is fundamental to developing the statistical theory [44,45,46]. The molecular mechanisms corresponding to rubber elasticity have been proposed in previous works on the statistical theories that explain the mechanical properties of rubbers [47,48,49]. These works developed a good basic understanding of rubber elasticity. Next, advanced theories were developed to explain the effect of entangled chains in rubber networks while still agreeing with the statistical theory of rubber elasticity [44,50,51]. However, we applied this theory for our rubber foams in compression mode. On the simplest level, we propose that the deformation in compression is accompanied by a decrease in entropy (*S*) with an increase in internal energy (*U*) of the molecular network. Figure 4, Figure 5, Figure 6 and Figure 7 present the evolution of compression force as a function of temperature for each compression strain of the control sample, control - 15% Po sample, control - 30% Po sample, and control - 45% Po sample, respectively. We found that the compression force of the rubber foam increases with increasing compression strain. The compression force also tends to decrease with increasing temperature at a given strain. Meanwhile, the compression force slope and the temperature curve tend to decrease at a higher compression strain because of a reduction of the degree of freedom of rubber molecules in the foam sample, which is related to the reduction of entropy. Thus, there is an entropy decrease on compression and an increase on retraction, except

i. at very low deformations, below about 10%, where a so–called thermoplastic inversion is observed due to thermal expansion obscuring the entropy effect [44];
ii. at large deformations, where high degrees of orientation and crystallisation may occur [33].

**Figure 8:**
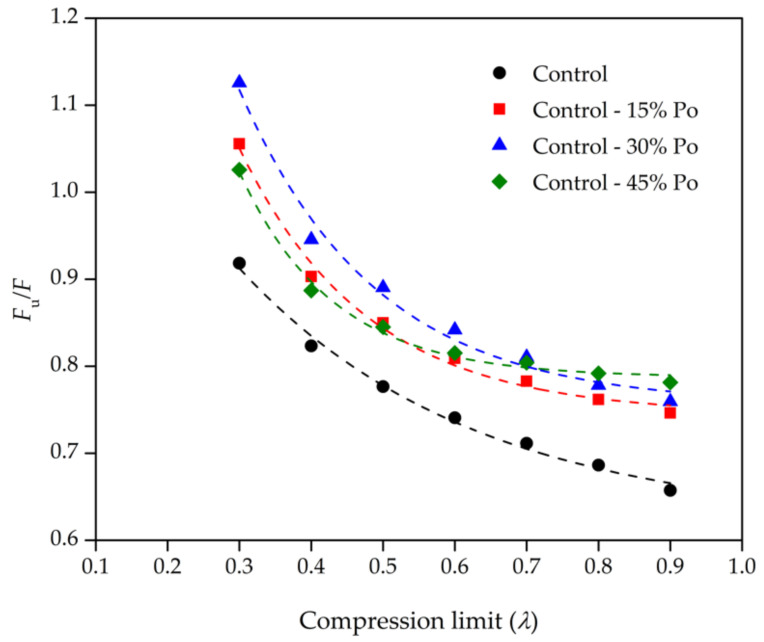
Ratios of the internal energy force (*F*_u_) to the compression force (*F*) as a function of the compression limit (*λ*) for various rubber foams. The scatter on the results is on the order of the size of the data points.

Based on the thermodynamics, the physical elasticity during the compression deformation of rubber molecules corresponds to the changes of internal energy force (*F*_u_) and entropy force (*F*_s_); thus, the compression force (*F*) is the summation of the internal energy force and the entropy force [44]. Table 5 shows that *F*_u_ and *F* for all the samples increase with increasing compression strain or decreasing compression limit, which is in reasonable agreement. However, the *F*_u_/*F* values of various crosslinked rubber foams in this study range from 0.7 to 1.2. From the perspective of the available literature, the *F*_u_/*F* values of the crosslinked natural rubber foam in the compression mode are in the range of 0.6–0.8 [33], while the foam density of that study was lower than that in our study. For the uncrosslinked solid natural rubber in extension mode, the *F*_u_/*F* values in the literature are in the range of 0.1–0.2 [44]. These different *F*_u_/*F* ratios should be different for different rubber networks, characterisation methods, and the formulas of compounded rubbers. After taking those factors into account, we can express the relationship between the *F*_u_/*F* ratios and the compression limit of rubber foam, as shown in Figure 8. This relationship is in good agreement with natural rubber foam reinforced by silica [33]. Our natural rubber foams with various concentrations of chemical blowing agent represent a similar degree of freedom of rubber molecules at different compression limits, indicating the stability of the degree of freedom of rubber chains at different compression limits.

## 4. Conclusions

Natural rubber foams were produced with different formulations by varying the chemical blowing agent concentration in the compounded latex. The morphological properties of rubber foam were very sensitive to the amount of the chemical blowing agent compared to the response of the physical elasticity. When the chemical blowing agent of rubber foam was decreased by 45%, the average cell size was also decreased to around 50%, while the cell density increased by around 800%. The average cell size, porosity and cell density of the rubber foams varied according to the concentration of the chemical blowing agent, whereas the compression of the rubber foams was similar for all samples. However, we measured a different mechanical property for the NR foam using the Mooney–Rivlin equation at low strain. Therefore, the mechanical properties of rubber foam depend on the morphological structure, the formulation of the compounded latex and the testing method. The ratio of the internal energy force (*F*_u_) to the compression force (*F*) of this work aligns with those in previous literature reviews. The chemical elasticity from crosslinked rubber networks can be used to calculate the thermodynamic parameters ∆*G* and ∆*S*. This study is a semi–empirical model for controlling the morphological properties of rubber foams to obtain different foam structures while retaining similar mechanical properties, especially compression. One can imagine tuning the structure of foam insulation or pillows to control the airflow to obtain the optimum mechanical property for the intended use.

## Figures and Tables

**Table 1:** Formulas of rubber foams at various chemical blowing agents: control sample, control sample with decreasing Po at 15% (control - 15% Po), control sample with decreasing Po at 30% (control - 30% Po), and control sample with decreasing Po at 45% (control - 45% Po).

Chemical Agents	Control (g)	Control - 15% Po (g)	Control - 30% Po (g)	Control - 45% Po (g)
60% concentrated natural latex	166.67	166.67	166.67	166.67
10% potassium oleate aqueous solution (Po)	16.50	14.00	11.50	9.00
50% sulphur aqueous dispersion	4.00	4.00	4.00	4.00
50% ZDEC aqueous dispersion	2.00	2.00	2.00	2.00
50% ZMBT aqueous dispersion	2.00	2.00	2.00	2.00
50% antioxidant (Wingstay L) aqueous dispersion	2.00	2.00	2.00	2.00
50% ZnO aqueous dispersion	10.00	10.00	10.00	10.00
33% DPG aqueous dispersion	2.00	2.00	2.00	2.00
12.5% SSF aqueous dispersion	8.00	8.00	8.00	8.00

**Table 2:** Morphological characteristics of various foam samples: cell size and porosity were determined by analysing the SEM images with ImageJ. The cell density was calculated using the same method in previous work [33].

Sample	Average Cell Size (±150 µm)	Porosity (±1.00%)	Cell Density (±500 cm^−^^3^)
Control	548	49.86	10,241
Control - 15% Po	473	54.73	15,908
Control - 30% Po	320	56.06	50,837
Control - 45% Po	273	54.78	82,450

**Table 3:** Constant values *C*_1_ and *C*_2_ from the Mooney–Rivlin equation [17] at different compression or extension limits (***λ***) of various rubber samples.

Type of Rubber	Test	*λ*	*C*_1_	*C*_2_	*C*_2_/*C*_1_	Reference
Crosslinked NR foam	Compression	<1	1.1809	0.1330	0.113	-
Uncrosslinked solid NR	Extension	1 ≤ *λ* ≤ 2	1.7725	2.7042	1.526	[42]
Crosslinked solid PDMS	Extension	< 1	2.91	1.98	0.682	[43]

**Table 4:** The volume fraction of rubber from the swelling test and thermodynamic parameters (Δ*G* and Δ*S*) from the Flory–Huggins equation [21,22].

Sample	Volume Fraction of Rubber (*V*_r_ ± 0.001%)	Δ*G* (J/mol)	Δ*S* (J/mol.K)
Control	0.2594	−36.3233	0.1218
Control - 15% Po	0.2574	−35.6039	0.1194
Control - 30% Po	0.2540	−34.3942	0.1154
Control - 45% Po	0.2524	−33.8061	0.1134

**Table 5:** The ratio of the internal energy force (*F*_u_) to the compression force (*F*) of various foam samples for different compression limits at 298.15 K.

Foam Sample	Compression Limit (*λ*)	*F*_u_ (±5% N)	*F* @ 298.15 K (±5% N)	*F*_u_/*F*
Control	0.9	6.35	9.66	0.6573
	0.8	8.61	12.55	0.6863
	0.7	10.37	14.57	0.7115
	0.6	12.52	16.91	0.7408
	0.5	15.66	20.16	0.7767
	0.4	21.68	26.34	0.8234
	0.3	33.20	36.15	0.9183
Control - 15% Po	0.9	4.56	6.11	0.7463
	0.8	6.48	8.51	0.7617
	0.7	8.16	10.43	0.7827
	0.6	10.36	12.81	0.8091
	0.5	13.32	15.68	0.8498
	0.4	18.89	20.91	0.9031
	0.3	28.30	26.81	1.0556
Control - 30% Po	0.9	3.72	4.85	0.7663
	0.8	5.39	6.91	0.7800
	0.7	6.88	8.52	0.8075
	0.6	8.99	10.69	0.8410
	0.5	11.90	13.27	0.8966
	0.4	17.46	17.97	0.9718
	0.3	26.81	22.31	1.2018
Control - 45% Po	0.9	4.26	5.46	0.7814
	0.8	6.24	7.88	0.7918
	0.7	8.20	10.20	0.8041
	0.6	10.91	13.39	0.8152
	0.5	14.47	17.12	0.8450
	0.4	20.85	23.51	0.8871
	0.3	30.91	30.14	1.0257

## Data Availability

The data that support the findings of this study are available from the corresponding author upon reasonable request.

## Supplementary Materials

The following are available online at https://www.mdpi.com/article/10.3390/polym13071091/s1, Table S1: Formulas of rubber foams at various chemical blowing agents, Figure S1: ATR–FTIR spectrum of control foam sample at 500–4000 cm^−1^, the other foam samples with reducing of chemical blowing agent represent the same ATR–FTIR spectrum with the control foam sample.
