# Supplementary material for: The Relationship between the Morphology and Elasticity of Natural Rubber Foam Based on the Concentration of the Chemical Blowing Agent
Source: Polymers (Basel). 2021 Mar 30;13(7):1091. doi: 10.3390/polym13071091 (PMC8036522; doi:10.3390/polym13071091)
Supplement: Supplementary file 1 [file polymers-13-01091-s001.pdf]

polymers

Supplementary Materials

The Relationship between the Morphology and Elasticity of Natural Rubber Foam Based on the Concentration of the Chemical Blowing Agent

Supitta Suethao [1], Saree Phongphanphanee [1], Jirasak Wong-ekkabut [1,2] and Wirasak Smitthipong [1,3,*]

[1] Specialized center of Rubber and Polymer Materials in agriculture and industry (RPM), Department of Materials Science, Faculty of Science, Kasetsart University, Bangkok, 10900, Thailand; supitta.sue@gmail.com (S.S.), fscisrph@ku.ac.th (S.P.), fscijsw@ku.ac.th (J.W.)
[2] Department of Physics, Faculty of Science, Kasetsart University, Bangkok, 10900, Thailand
[3] Office of Research Integration on Target-based Natural Rubber, National Research Council of Thailand (NRCT), Bangkok, 10900, Thailand
* Correspondence: fsciwssm@ku.ac.th

Keywords: rubber foam; morphology; elasticity; thermodynamics; chemical blowing agent

Table S1. Formulas of rubber foams at various chemical blowing agents.

Chemical agents	Formulas (phr[1])			
	Control	Control - 15% Po	Control - 30% Po	Control - 45% Po
60% concentrated natural latex	100.00	100.00	100.00	100.00
10% potassium oleate aqueous solution (Po)	1.65	1.40	1.15	0.90
50% sulphur aqueous dispersion	2.00	2.00	2.00	2.00
50% ZDEC aqueous dispersion	1.00	1.00	1.00	1.00
50% ZMBT aqueous dispersion	1.00	1.00	1.00	1.00
50% antioxidant (Wingstay L) aqueous dispersion	1.00	1.00	1.00	1.00
50% ZnO aqueous dispersion	5.00	5.00	5.00	5.00
33% DPG aqueous dispersion	0.66	0.66	0.66	0.66
12.5% SSF aqueous dispersion	1.00	1.00	1.00	1.00

[1] Parts per hundred of rubber.

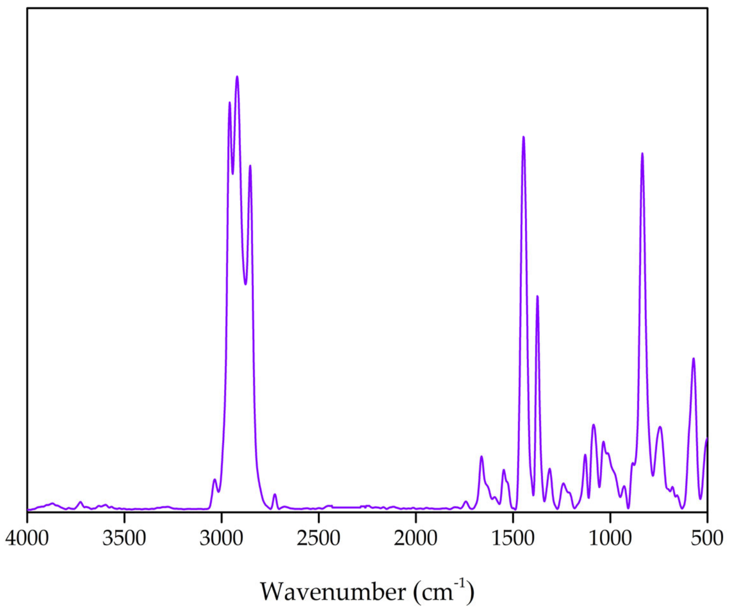

Figure S1. ATR–FTIR spectrum of the control foam sample at 500–4000 cm^{-1}, the other foam samples with reducing of the chemical blowing agent represent the same ATR–FTIR spectrum with the control foam sample.
